# Causal relationship of familial hypercholesterolemia with risk of intestinal vascular disorders: A mendelian randomization study

**DOI:** 10.1016/j.metop.2025.100352

**Published:** 2025-01-31

**Authors:** Gang Wei, Cheng Zhang, Feng-Jie Shen, Hua-Qi Guo, Lin Liu

**Affiliations:** a Beijing Key Laboratory of Diabetes Research and Care, Department of Endocrinology, Beijing Diabetes Institute, Beijing Tongren Hospital, Capital Medical University, Beijing, 100730, China; b Key Laboratory of Pollution Exposure and Health Intervention of Zhejiang Province, Hangzhou, 310015, China; c Department of Pulmonary and Critical Care Medicine, The Ninth People's Hospital of Shanghai Jiao Tong University School of Medicine, Shanghai, 200011, China

**Keywords:** Familial hypercholesterolemia, Intestinal vascular diseases, *ApoB*, *PCSK9*, *CDKN2B-AS1*

## Abstract

**Background:**

The causal relationship between the familial hypercholesterolemia (FH) and intestinal vascular diseases was unnoticed. This study aims to investigate the cause-and-effect relationship of FH with risk of intestinal vascular diseases in human.

**Methods:**

A Mendelian randomization (MR) analysis was performed by extracting summary-level datasets for FH or FH concurrently with ischemic heart disease (IHD) and intestinal vascular diseases from the FinnGen study including 329,115, 316,290 and 350,505 individuals. The inverse-variance weighted (IVW) method and the weighted median method were applied to analyze the causal relationships between FH or FH concurrently with IHD and the risk of intestinal vascular diseases. Cochran's Q statistic method and MR-Egger regression were used to assess heterogeneity and pleiotropy.

**Results:**

The IVW method demonstrated that FH was significantly associated with higher odds of intestinal vascular diseases [OR (95%CI): 1.22 (1.03, 1.45)] (*P* = 0.02) without significant heterogeneity (*P* = 0.54) and horizontal pleiotropy (*P* = 0.43). Rs7575840 in 6.5kda upstream of *ApoB* gene, rs11591147 in *PCSK9* gene and rs9644862 in the *CDKN2B-AS1* (or named *ANRIL; p15AS*; *PCAT12*; *CDKN2BAS*; *CDKN2B-AS*; *NCRNA00089*) gene were illustrated to mostly influence the risk of intestinal vascular diseases. However, no significant causal relationship between FH concurrently with IHD and intestinal vascular diseases was observed.

**Conclusion:**

In conclusion, FH was causally positive-associated with the increased risk of intestinal vascular diseases, revealing a potential unfortunate outcome for FH. Therefore, patients with FH should pay closely attention to the risk of intestinal vascular diseases. Our study may provide evidence for new diagnostic and therapeutic strategies in clinical practices.

## Highlights

• FH was significantly associated with higher odds of intestinal vascular diseases.
• The causal link of FH and intestinal vascular disease was solid without significant heterogeneity and horizontal pleiotropy.
• The risk of intestinal vascular disease was associated with *ApoB*, *PCSK9* and *CDKN2B-AS1*.
• No significant causal relationship was found between FH coupled with IHD and intestinal vascular diseases.

## Graphical abstract

**Figure:**
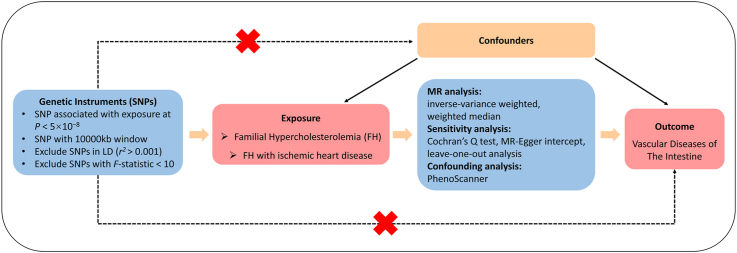

## Key messages

### What is already known on this topic?

Patients with familial hypercholesterolemia (FH) may have intestinal vascular diseases, while their causal relationships were still unclear.

### What this study adds?

This study aimed to investigate the cause-and-effect relationship of FH with risk of intestinal vascular diseases in human. FH was causally positive-associated with the increase risk of intestinal vascular diseases, revealing a potential unfortunate outcome for FH.

How this study might affect research, practice or policy?

Our study may provide evidence for new diagnostic and therapeutic strategies in clinical practices.

## 1 Introduction

Intestinal vascular diseases are ischemia lesions of intestinal blood vessels with the frequent pathogenesis of insufficient blood flow in specific section of intestine, which induces ischemia, inflammation, disruption of intestinal mucosal permeability, perforation, and ultimately irreversible and catastrophic tissue damage, such as necrosis [[1], [2], [3]]. The common clinical manifestations of intestinal vascular diseases include acute mesenteric ischemia (AMI), chronic mesenteric ischemia (CMI) (also called intestinal angina), mesenteric venous thrombosis (MVT) and ischemic colitis, etc [4]. The death rate of intestinal vascular diseases ranges from 30 % to 90 % according to different causes [[4], [5], [6]]. However, to date, the exact diagnosis of intestinal vascular diseases remains challenging, which make it difficult for clinical practice application [1].

Familial hypercholesterolemia (FH) is the autosomal dominant or codominant metabolic defect inherited from parents with the characteristic of high plasma level of low-density lipoprotein cholesterol (LDL-C) [[7], [8], [9], [10]]. Abnormal LDL-C concentrates in blood have been found to be implicated in several vascular diseases, including intestinal vascular diseases [11]. The gut-vascular barrier (GVB) is a functional/anatomical structure mainly formed by endothelial vascular and intestinal cells. Abnormalities of intestinal mucosal microvasculature, which is located underneath the intestinal epithelial layer, dysregulates the passage of (macro) molecules across GVB [12,13]. Typically, in the absence of diagnosis and proper treatment, lifelong hyper-concentration of blood LDL-C accumulates the premature formation of atherosclerosis and subsequent vulnerability to intestinal vascular diseases [4,14,15]. In this situation, the patients with FH are at risk of suffering from intestinal vascular diseases throughout their whole course of disease. However, there is no published direct genetic-evidence manifesting whether the patients with FH will develop intestinal vascular diseases during their lifetime.

Given its challenging diagnosis, intestinal vascular diseases have nowadays been the point of increasing interest in clinic practice. Therefore, it is imperative to study the predictive genetic-fashion of FH on the risk of intestinal vascular diseases to prevent the development of intestinal vascular diseases under the condition of FH. In this study, we performed a Mendelian randomization (MR) design to assess possible causal relationship of FH with risk of intestinal vascular diseases. In the present study, we demonstrated that FH but not FH concurrently with IHD was causally positive-associated with the increased risk of intestinal vascular diseases [OR (95%CI): 1.22 (1.03, 1.45)] (*P* = 0.02). Moreover, we revealed that rs7575840 in *ApoB* gene, rs11591147 in *PCSK9* gene and rs9644862 in *CDKN2B-AS1* gene were closely related to the risk of intestinal vascular diseases. Our findings provide evidence for new diagnostic and therapeutic strategies in clinical practices.

## 2 Materials and methods

### 2.1 Mendelian randomization (MR) design

**Fig. 1:**
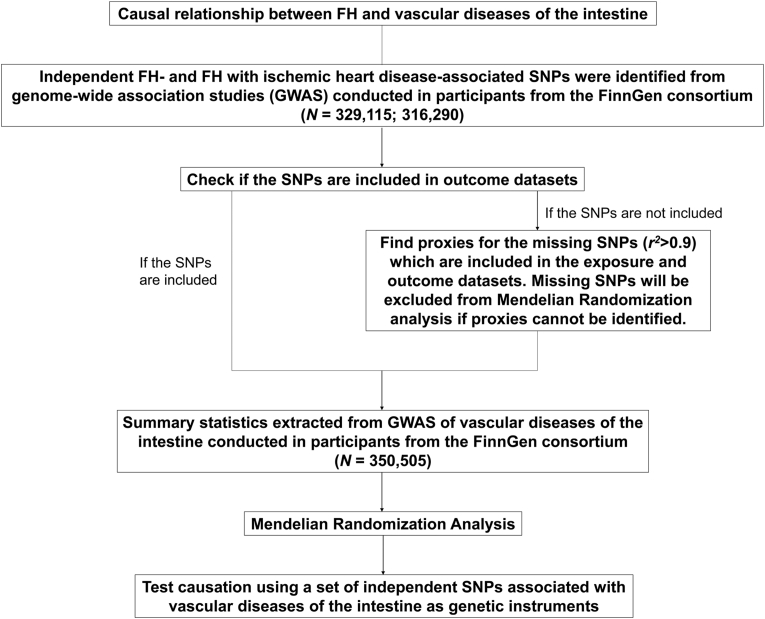
Flowchart of the Mendelian randomization analyses of familial hypercholesterolemia and risk of intestinal vascular diseases. FH, familial hypercholesterolemia.

For MR analysis, the inclusion of instrumental variables follow three primary assumptions [16,17]. A MR design based on public summary-level data was derived from genome-wide association studies (GWASs), which was adopted to evaluate the possible causal relationship of FH and FH concurrently with ischemic heart disease (IHD) to the risk of intestinal vascular diseases. An overview of the study design was shown in Fig. 1.

### 2.2 Instrumental variable selection

The analyses were based on publicly available data that have been approved by relevant review boards. The genetic variants of FH and FH concurrently with IHD were from the recently published GWAS of European ancestry (FinnGen) (N = 329,115; 316,290). The FinnGen was approved by the Coordinating Ethics Committee of the Hospital District of Helsinki and Uusimaa (HUS/990/2017). The summary datasets are available on the website (https://storage.googleapis.com/finngen-public-data-r9/summary_stats/finngen_R9_E4_FH.gz; https://storage.googleapis.com/finngen-public-data-r9/summary_stats/finngen_R9_E4_FH_IHD.gz). Single nucleotide polymorphisms (SNPs) associated with FH, and FH concurrently with IHD were selected at the genome-wide significance level of *P* < 5 × 10^−8^. When the *r*^*2*^ of two SNPs was less than 0.001, the SNP with a higher significant *P*-value was removed from the analysis. SNPs not available in the datasets of intestinal vascular diseases were either replaced with proxy SNPs in high linkage disequilibrium (*r*^*2*^ > 0.9) or discarded. Finally, palindromic SNPs may be discarded based on their allele frequencies.

### 2.3 Outcome data source

The genetic variants of intestinal vascular diseases were from the recently published GWAS of European ancestry (FinnGen) (*N* = 350,505). The summary dataset is available on the website (https://storage.googleapis.com/finngen-public-data-r9/summary_stats/finngen_R9_I9_VASCINT.gz).

### 2.4 Mendelian randomization methods

The MR method was used to explore the causal associations of genetically predicted difference of the per one standard deviation (SD) increase in FH with the risk of intestinal vascular diseases by reporting odds ratios (ORs). Importantly, the conventional inverse-variance weighted (IVW) method and the weighted median (WM) method were used in the primary analysis [18]. The Cochran's Q test under the IVW model was used to quantify heterogeneity [19]. The MR-Egger regression model was used to check for unknown pleiotropic effects. A non-zero intercept from MR–Egger indicates that the IVW estimate may be invalid due to horizontal pleiotropy [20]. Sensitivity analysis based on the MR-Egger regression model and leave-one-out sensitivity analysis were also performed [21]. Furthermore, the PhenoScanner database was searched to see whether the selected SNPs were associated with any potential confounders, at a genome-wide significance level of *P* < 5 × 10^−8^ [22,23].

### 2.5 Statistical analyses

All statistical analyses were conducted by R software (R 4.0.5, The R Foundation for Statistical Computing) and “TwoSampleMR” package [24]. The two-sided significance level was set at 0.05 for all analyses.

## 3 Results

### 3.1 The genetic variants associated with familial hypercholesterolemia (FH)

As the design of Mendelian randomization (MR), we collected the genetic variants of FH, and FH concurrently with IHD by analyszing the recently published GWAS of European ancestry (FinnGen) (N = 329,115; 316,290). Detailed information has been shown in Fig. 1 and Table 1. In the instrumental variable, we found that 13 SNPs associated with FH, and 10 SNPs associated with FH concurrently with IHD were obtained (each *P* < 5 × 10^−8^, *r*^*2*^ < 0.001) (Table 2, Table 3, respectively). These results indicated that the genetic variants were closely related to FH, which can be further used for investigating the potential cause-and-effect association of FH with intestinal vascular diseases.

**Table 1:** Description of included datasets definition in FinnGen consortium derived from ICD codes.

Exposure/Outcome	No. of cases/controls	ICD-10 diagnosis	ICD-9 diagnosis	ICD-8 diagnosis	ICD-10 exclusion	ICD-9 exclusion	ICD-8 exclusion
FH	4965/324150	E7800	2720A	$!$			
FH concurrently with IHD	2757/313533						
Intestinal vascular diseases	966/349539	K55 [0–1]|K559	557	4442			

**Abbreviations**: ICD, International Classification of Disease.

**Table 2:** Characteristics of genetic variants associated with FH used in the Mendelian randomization analysis.

SNP	Chr	Position	Nearest gene(s)	Effect allele	Other allele	EAF	*Β* estimate^a^	SE	*P*-value	Proxy SNP	r^2^
rs11591147	1	55505647	PCSK9	T	G	0.037087	−0.50812	0.06264	4.99E-16	NA	1.00
rs499883	1	55519174	PCSK9	A	G	0.526842	0.144529	0.020575	2.15E-12	NA	1.00
rs646776	1	109818530	CELSR2	T	C	0.783898	0.171424	0.025445	1.62E-11	NA	1.00
rs7575840	2	21273490	APOB	T	G	0.292212	0.122598	0.022067	2.76E-08	NA	1.00
rs185567543	4	74990001	MTHFD2L	T	A	0.021341	0.36879	0.06461	1.14E-08	NA	1.00
rs118039278	6	160985526	LPA	A	G	0.046116	0.250891	0.045168	2.78E-08	NA	1.00
rs9644862	9	22090936	CDKN2B-AS1	G	T	0.433327	0.115318	0.020464	1.75E-08	NA	1.00
rs115478735	9	136149711	–	T	A	0.197663	0.141933	0.0249	1.20E-08	NA	1.00
rs964184	11	116648917	ZPR1	C	G	0.856689	−0.23882	0.027606	5.11E-18	NA	1.00
rs112898275	19	11188850	LDLR	C	T	0.102943	−0.32103	0.036196	7.36E-19	NA	1.00
rs143466522	19	11318472	DOCK6	A	G	0.014489	0.560841	0.073252	1.91E-14	NA	1.00
rs142834163	19	11381898	DOCK6	A	G	0.013024	0.508043	0.078509	9.72E-11	NA	1.00
rs7412	19	45412079	APOE	T	C	0.054518	−0.42272	0.049159	8.05E-18	NA	1.00

a The *β* estimates are defined for each additional effect allele. Positive *β* indicates more frequency of fungal infection. **Abbreviations**: Chr, chromosome; EAF, effect allele frequency; SNP, single nucleotide polymorphism; SE, standard error.

**Table 3:** Characteristics of genetic variants associated with FH concurrently IHD used in the Mendelian randomization analysis.

SNP	Chr	Position	Nearest gene(s)	Effect allele	Other allele	EAF	*Β* estimate^a^	SE	*P*-value	Proxy SNP	r^2^
rs11591147	1	55505647	PCSK9	T	G	0.03686	−0.66464	0.089021	8.26E-14	NA	1.00
rs499883	1	55519174	PCSK9	A	G	0.527281	0.179182	0.028314	2.48E-10	NA	1.00
rs646776	1	109818530	CELSR2	T	C	0.784147	0.226547	0.035351	1.47E-10	NA	1.00
rs118039278	6	160985526	LPA	A	G	0.045379	0.337893	0.061645	4.22E-08	NA	1.00
rs2286427	7	76024520	SSC4D	T	C	0.136412	0.218547	0.039108	2.29E-08	NA	1.00
rs9644860	9	22090603	CDKN2B-AS1	T	C	0.41375	0.233478	0.028109	9.88E-17	NA	1.00
rs964184	11	116648917	ZPR1	C	G	0.856021	−0.24324	0.037986	1.52E-10	NA	1.00
rs59697303	15	79071961	ADAMTS7	T	C	0.363772	0.166484	0.028939	8.77E-09	NA	1.00
rs73015011	19	11189764	LDLR	C	T	0.102961	−0.3292	0.049948	4.37E-11	NA	1.00
rs7412	19	45412079	APOE	T	C	0.05402	−0.46308	0.068013	9.85E-12	NA	1.00

a The *β* estimates are defined for each additional effect allele. Positive *β* indicates more frequency of fungal infection. **Abbreviations**: Chr, chromosome; EAF, effect allele frequency; SNP, single nucleotide polymorphism; SE, standard error.

### 3.2 Cause-and-effect relationship of FH with intestinal vascular diseases

According to the inverse-variance weighted (IVW) model, as shown in Table 4, FH was significantly associated with higher odds of intestinal vascular diseases [OR (95 % CI): 1.22 (1.03, 1.45)] (*P* = 0.02). However, there was no causal relationship of FH concurrently with IHD on risk of intestinal vascular diseases (*P* > 0.05) (Table 4). To investigate whether the original MR analyses were robust to the violations of assumptions, we further evaluated the heterogeneity and pleiotropy of the results by using Cochran's Q statistic method and MR-Egger regression. As displayed in Table 2, no significant heterogeneity (*P* = 0.54) and horizontal pleiotropy (*P* = 0.43) were presented in the correlation analysis of FH and intestinal vascular diseases (Table 5). These results indicated that the genetic variants were involved in the risk of FH-triggered intestinal vascular diseases.

**Table 4:** Inverse variance-weighted (IVW) and weighted median analyses of familial hypercholesterolemia (FH) and risk of intestinal vascular diseases.

Exposure	Inverse variance-weighted method		Weighted median method			
	SNPs, n	OR (95%CI)	*P*-value	SNPs, n	OR (95%CI)	*P*-value
FH	13	1.22 (1.03, 1.45)	0.02	13	1.19 (0.94, 1.49)	0.14
FH concurrently with IHD	10	1.14 (0.99, 1.33)	0.08	10	1.16 (0.96, 1.42)	0.13

**Table 5:** Cochran's Q test, MR–Egger intercept and MR-Egger regression of familial hypercholesterolemia (FH) and risk of intestinal vascular diseases.

Exposure	Cochran's Q test^a^	MR-Egger intercept^b^			MR-Egger regression			
	*P*-value	*β*	SE	*P*-value	SNPs, n	*β*	SE	*P*-value
FH	0.54	0.03	0.04	0.43	13	0.07	0.18	0.73
FH concurrently with IHD	0.57	0.03	0.05	0.60	10	0.24	0.21	0.28

a The Cochran's Q test is a statistical test for heterogeneity.

b The intercept term from the MR–Egger regression method is a statistical test of horizontal pleiotropy.

### 3.3 Result of leave-one-out sensitivity analysis

**Fig. 2:**
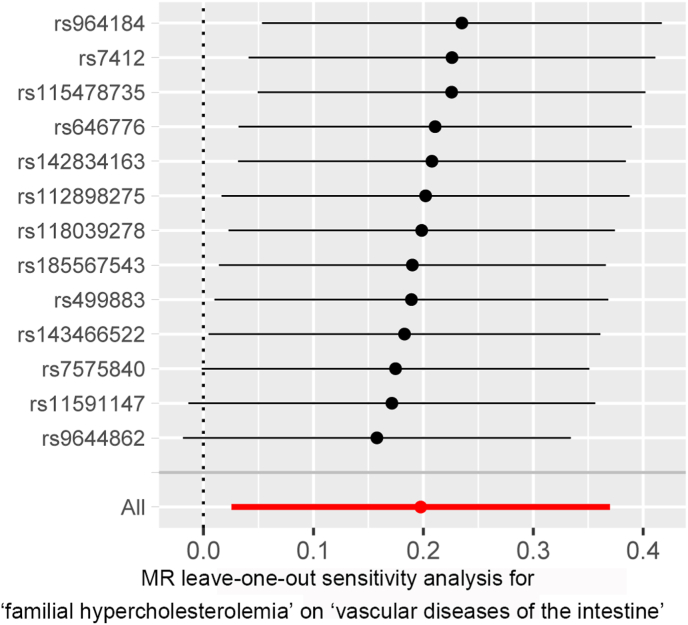
Leave-one-out sensitivity and MR analyses based on the inverse-variance weighted (IVW) model for determining the effects of familial hypercholesterolemia on risk of intestinal vascular diseases.

In the leave-one-out sensitivity analysis, by leaving rs7575840, rs11591147 and rs9644862 out separately, the correlation of FH with intestinal vascular diseases remained, showing a strong robustness of the observed result (*β* = 0.122598; −0.50812; 0.115318) (Fig. 2 and Table 2). Therefore, these results revealed that rs7575840 in *ApoB* gene, rs11591147 in *PCSK9* gene and rs9644862 in *CDKN2B-AS1* gene were closely related to the risk of intestinal vascular diseases.

### 3.4 The mechanistic model for the potential cause-and-effect relationship

**Fig. 3:**
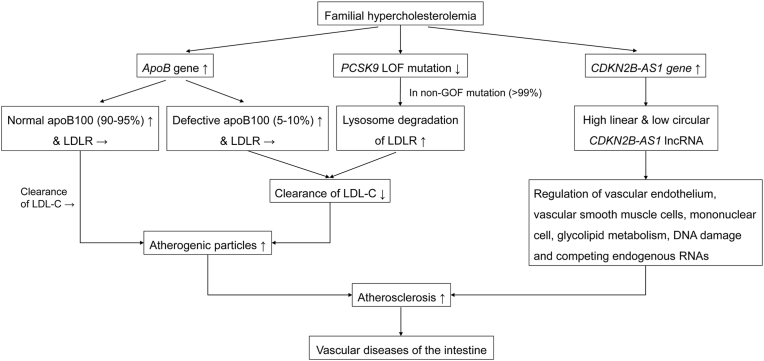
**Representing signaling pathways involved in the associations between familial hypercholesterolemia and intestinal vascular diseases.** Abbreviations:*ApoB*, apolipoprotein B; *PCSK9*, proprotein convertase subtilisin/kexin 9; *CDKN2B-AS1*, cyclin-dependent kinase inhibitor 2B antisense RNA1; LOF, loss of function; GOF, gain of function; *LDLR*, low-density lipoprotein receptor.

Given the solid and reliable causal effect of FH on the development of intestinal vascular diseases as demonstrated by MR analysis and subsequent leave-one-out sensitivity analysis, we proposed a mechanistic model that exhibited the cause-and-effect relationship between FH and intestinal vascular diseases. These dependencies for the mechanistic model that exhibited the potential cause-and-effect relationship of FH and intestinal vascular diseases are displayed in Fig. 3.

## 4 Discussion

In the present study, we evaluated the causal effect of FH on the development of intestinal vascular diseases for the first time. The MR analysis suggested that FH was significantly associated with increased odds of intestinal vascular diseases, showing an underlying adverse effect of FH on the risk of intestinal vascular diseases. The conclusion was further supported by leave-one-out sensitivity analysis. Specifically, we revealed that rs7575840 in *ApoB* gene, rs11591147 in *PCSK9* gene and rs9644862 in *CDKN2B-AS1* gene were closely related to the risk of intestinal vascular diseases. On this basis, we proposed a mechanistic model that exhibited the cause-and-effect relationship between FH and intestinal vascular diseases as mentioned above (Fig. 3).

In the MR analysis, the genetic variants of FH or FH concurrently with IHD and intestinal vascular diseases were from the recently published GWAS of European ancestry (FinnGen) [25]. In the instrumental variable of exposure, we defined that 13 SNPs that were closely associated with FH, whereas 10 SNPs were related to FH concurrently with IHD through clumping analysis (each *P* < 5 × 10^−8^, *r*^*2*^ < 0.001). In further IVW analysis, we found that FH was positively associate with the risk of intestinal vascular diseases. Moreover, we did not found obvious differences in both heterogeneity and horizontal pleiotropy for this significant association, suggesting that the original MR assumptions were not violated. To elucidate the hidden mode of action behind the discovery of the MR analysis, the leave-one-out sensitivity analysis was performed. By leaving rs7575840, rs11591147 and rs9644862 out, respectively, the results for the correlation of FH with risk of intestinal vascular diseases did not change, indicating robust and reliable results in the analysis. These result indicated that rs7575840 in *ApoB* gene, rs11591147 in *PCSK9* gene and rs9644862 in *CDKN2B-AS1* gene were identified as the major genetic variant associated with FH, which probably contributed to the risk of intestinal vascular diseases. Typically, the *β*-coefficients of rs7575840 and rs9644862 were positive, while rs11591147 was negative.

Rs7575840 is an SNP in 6.5 kb upstream of apolipoprotein B (*ApoB*) gene [26]. Rs7575840 positively manipulates the expression of *ApoB* gene through affecting a transcription factor binding site (TFBS) of CEBPA [26]. The *ApoB* gene is 43 kb on the short arm of chromosome 2 and contains 29 exons [27]. The *ApoB* gene encodes protein of apoB in two isoforms involving apoB48 produced by small intestine and apoB100 produced by liver [28]. The molecular weight of apoB100 is 540 kDa, and the apoB48 accounting for 48 % of it [28]. The two types of apoB have completely different physiological functions. ApoB48 possesses 2512 amino acids (265 kDa) and is a component of chylomicrons with the ratio of one apoB48 molecule per particle, contributing to intestinal uptake of dietary fat [28,29]. The apoB100 protein contains 4536 amino acids (550 kDa) and essentially implicates in the hepatic assembly of very low-density lipoprotein (VLDL-C). of note, VLDL-C will be metabolized to intermediate-density lipoprotein (IDL-C) and then into LDL-C [29]. After LDL-C catabolized from VLDL-C, apoB100 on the LDL-C surface can bind the LDL-C receptor (LDLR) and mediate the elimination of blood LDL particles [29]. The primary binding region (also called site B) that interacts with LDLR is mainly located at residues 3356–3368 of apoB [29]. All the above lipoproteins are atherogenic or latent-atherogenic particles with the ratio of one apoB100 molecule in each particle, which implies that apoB directly reflects the amount of circulating atherogenic particles that accelerate the formation of intra-arterial plaques [28,29].

In all mutations of FH, the mutation in *ApoB* gene accounts for 5%–10 % [30]. The missense in *ApoB* gene is a causative variant that reduces the adhesive ability of apoB100 to LDLR, resulting in a declined clearance of LDL-C [30]. The first and most active alteration in the key mutation region of exon 26 in *ApoB* gene is a single amino acid replacement of arginine with glutamine at position 3527 p. (Arg3527Gln), followed by later finding of p. (Arg3527Trp), p. (Arg3527Leu), p. (Arg3507Trp) and p. (Trp4396Tyr) [31]. Functionally, p. (Arg3527Gln), p. (Arg3527Trp) and p. (Arg3527Leu) have the capacity to shift the conformation of apoB100 on the surface of LDL-C, and p. (Arg3507Trp) and p. (Trp4396Tyr) can change the residues required for the binding of apoB100 to LDLR [31]. In addition, p. (Arg50Trp) in exon 3, p. (Arg3059Cys), p. (Lys3394Asn), p. (Arg1164Thr) and p. (Gln4494del) are other untraditional changes in *ApoB* gene [31]. When the expression of rs7575840 is elevated in FH patients with abnormal *ApoB* gene, the apoB100 with defective affinity to bind LDLR could be increased correspondingly, making lack of sufficient ligand of LDLR for LDL-C clearance. If the expression of rs7575840 is elevated in FH patients with unmutated *ApoB* gene, it will generate more regular apoB100 equal to the large quantity of atherogenic particles in circulation, but not a corresponding increase in the expression of LDLR. As a result, intestinal vascular diseases will occur due to the accumulation of atherosclerosis in the vascular of intestine [14].

Rs11591147 (137G > T, R46L) is an SNP in the proprotein convertase subtilisin/kexin 9 (*PCSK9*) gene [32]. The *PCSK9* gene is 25 kb on the small arm of chromosome 1p32 and consists of 12 exons and 11 introns [33]. The secreted protein of PCSK9 encoded by *PCSK9* gene primarily comes from liver, with less from kidney and intestine [33]. The proprotein of PCSK9 is initially synthesized as inactive, which then gets into the endoplasmic reticulum (ER) to start the autocatalytic cleavage of 692-amino acid peptide at VFAQ152–SIP position [33,34]. The mature PCSK9 produced from cleavage process is transported from ER into Golgi by eukaryotic cells [33]. It is thereafter released into the circulation, where the catalytic domain (amino acids 153–421) of mature PCSK9 protein binds to the epidermal growth factor (EGF)-A domain (position 314–355) of the LDLR on hepatocellular surface (major) and in trans Golgi network (minor) by protein-protein crosstalk [33,35]. The LDLR/PCSK9 complex passes through the endosome into lysosome for degradation, which makes less LDLR return to cell membrane for cleaning LDL-C [31]. The mutation in *PCSK9* gene operates in two opposite ways, namely loss-of-function (LOF) causing hypocholesterolemia and gain-of-function (GOF) causing hypercholesterolemia [36]. In all mutations of FH, the GOF-mutation in *PCSK9* gene accounts for <1 %, so the remaining >99 % belongs to non-GOF [30]. The rs11591147 (p. Arg46Leu) of *PCSK9* in exon 1 is believed as a LOF-mutation, which plays against hyper LDL-C by suppressing the linking ability of PCSK9 to LDLR, leading to a attenuation of lysosome-mediated LDLR degradation [31,32,37]. It has been indicated that p. (Arg46Leu) could reduce PCSK9 levels by 15 % [33,34]. Once the expression of *PCSK9* rs11591147 is inhibited in FH patients with this targeted variant, the connective capacity of PCSK9 to LDLR augments accordingly, which will facilitate the lysosome-mediated degradation of LDLR and thus lower the concentration of recycling LDLR. Therefore, intestinal vascular diseases of will onset because of the aggravation of atherosclerosis in the vascular of intestine, and badly short of LDLR for elimination of LDL-C [14,31].

Rs9644862 is an SNP in the cyclin-dependent kinase inhibitor 2B antisense RNA1 (*CDKN2B-AS1*) (or named antisense noncoding RNA in the INK4 locus (*ANRIL*); *p15AS*; *PCAT12*; *CDKN2BAS*; *CDKN2B-AS*; *NCRNA00089*) gene [[38], [39], [40]]. The CDKN2B-AS1 encoded by *CDKN2B-AS1* gene is a 3.8 kb long non-coding RNA (lncRNA) transcribed from the short arm of human chromosome 9p21.3 and takes 21 exons [41,42]. The CDKN2B-AS1 lncRNA has been detected to express in vascular endothelia cells (VECs), vascular smooth muscle cells (VSMCs), mononuclear phagocytes and atherosclerotic plaques [43]. The whole chain of CDKN2B-AS1 lncRNA could be spliced by RNA polymerase Ⅱ into linear (atherogenic) and circular (antiatherogenic) subtypes [42]. High linear and low circular CDKN2B-AS1 lncRNA will lead to atherosclerosis [42]. The mistaken phenotype of CDKN2B-AS1 lncRNA contributes to atherosclerosis by several approaches, including the vascular endothelium injury, proliferation/migration/senescence/apoptosis of VSMC, mononuclear cell adhesion, and proliferation, glycolipid metabolism disorder, DNA damage and competing endogenous RNAs (CERNA) [43]. The manners in vascular endothelium injury are attributed to the activation of CDKN2B-AS1/YY1‐IL6/8 pathway and the increase in the expression of caspase recruitment domain‐containing protein 8 (CARD8) and vascular endothelial growth factor (VEGF) [[43], [44], [45]]. The CDKN2B-AS1 depletion and its recruitment of SUZ12 (a core component of the PRC2 complex) and chromodomain of chromobox homolog 7 (CBX7, a subunit of PRC1) to INK4b‐ARF‐INK4a gene cluster have been reported to contribute to the proliferation/migration/senescence/apoptosis of VSMC [43]. CDKN2B-AS1 (with Alu elements) favors the adhesion and proliferation of mononuclear cell. Moreover, CDKN2B-AS1 interrupts glycolipid metabolism by ordering the expression of *ADIPOR1*, *VAMP3*, *C11ORF10*, endothelin‐1 (*ET‐1*), p16^INK4a^‐cdk4‐p‐RB axis, *CDKN2A* methylation and *NF‐κB* signaling pathway. CDKN2B-AS1 manipulates the expression of p14, p15 and p16 in INK4a‐ARF‐INK4b gene cluster during DNA damage. CDKN2B-AS1 stimulates cycle‐dependent kinases 6 (CDK6) to exert the role of CERNA by downregulating miR-449a [43]. Notably, the *CDKN2B-AS1* gene has not been reported to be a specific error allele for FH. Suppose the linear *CDKN2B-AS1* lncRNA is overexpressed exceeding circular *CDKN2B-AS1* lncRNA in FH patients with high display of *CDKN2B-AS1* rs9644862 gene, the atherosclerosis independent of the stimulation of inherited mutant gene will directly ascend. Thus, intestinal vascular diseases will origin from the mounting atherosclerosis in the vascular of intestine [14].

This study has several advantages. First, this is the first population-study to analyze the cause-and-effect relationships between FH and risk of intestinal vascular diseases. Second, the results were high believable because the genetic variants of FH and intestinal vascular diseases were sequenced from enormous population. Third, by leave-one-out sensitivity analysis, the causal relationships between FH and risk of intestinal vascular diseases was confirmed and the SNPs severely influencing the risk of intestinal vascular diseases in FH were found in this study. Nevertheless, there are also several limitations. First, the morbidity and survival expectation of intestinal vascular diseases in FH individuals could not be calculated in this MR study. Continued follow-up investigations of epidemiology will be warranted in prospective cohort study. Second, the pathways of intestinal vascular diseases encompass multiple molecular orchestrate [4]. However, the mechanism of FH on intestinal vascular diseases was only verified by leave-one-out sensitivity analysis. The undiscovered risk factors will be examined by more inspection methods in vitro and in vivo. Third, the LDL-C is closely correlated to IHD, the OR of which in those carrying FH mutant is 2.67 [95%CI: 1.71, 4.01]-fold higher than noncarriers. However, to date, no significant causal relationship between FH with IHD and risk of intestinal vascular diseases was observed. The worthwhile finding will be inquired in the future. Lastly, the most common mutation in FH, *LDLR*, did not perform indispensable role on intestinal vascular diseases. The mechanism distinct from *ApoB* and *PCSK9* will be inferred in the future. Nevertheless, our data only provide information associated with European regions, further parallel research is needed in other regions such as Asia-Pacific region. Besides, the future research needs to pay more attention to FH associated intestinal vascular diseases by directly comparing the difference between Europe and the rest of the world.

## 5 Conclusions

In the present study, our results indicated that FH was causally positive-associated with the increase risk of intestinal vascular diseases, revealing a potential unfortunate outcome for FH. In our results, we identified three major genetic variant associated with FH, including rs7575840 in *ApoB* gene, rs11591147 in *PCSK9* gene and rs9644862 in *CDKN2B-AS1* gene. These genetic variant in patients with FH may probably contribute to the development of intestinal vascular diseases. Therefore, patients with FH should pay closely attention to the risk of intestinal vascular diseases, and if necessary, such patients should receive targeted prevention and treatment for FH-induced intestinal vascular diseases.

## CRediT authorship contribution statement

**Gang Wei:** Writing – review & editing, Writing – original draft, Validation, Methodology, Funding acquisition, Conceptualization. **Cheng Zhang:** Writing – review & editing, Writing – original draft, Validation, Resources, Data curation. **Feng-Jie Shen:** Writing – original draft, Resources, Investigation, Data curation. **Hua-Qi Guo:** Writing – original draft, Software, Formal analysis, Conceptualization. **Lin Liu:** Writing – review & editing, Writing – original draft, Validation, Resources, Project administration, Methodology, Formal analysis, Conceptualization.

## Data availability statement

The summary datasets of FH or FH concurrently with IHD and intestinal vascular diseases are available on the websites (https://storage.googleapis.com/finngen-public-data-r9/summary_stats/finngen_R9_E4_FH.gz; ttps://storage.googleapis.com/finngen.

-public-data-r9/summary_stats/finngen_R9_E4_FH_IHD.gz; https://storage.googleap-is.com/finngen-public-data-r9/summary_stats/finngen_R9_I9_VASCINT.gz). Further inquiries can be directed to the corresponding authors.

## Ethics statement

Ethical approvals and consent information for participants of GWAS were collected from the original publications. New ethics approval and consent to participate were not required.

## Funding statement

This research was funded by the 10.13039/501100012166National Key Research and Development Program of China (2022YFA0807000), the 10.13039/501100001809National Natural Science Foundation of China (Nos. 82370547, 82000804), the Key Laboratory of Environmental Pollution Monitoring and Disease Control, Ministry of Education, 10.13039/501100010265Guizhou Medical University (No.440), Beijing Hospitals Authority Innovation Studio of Young Staff Funding Support (202305).

## Conflict interest

The authors declare that they have no known competing financial interests or personal relationships that could have appeared to influence the work reported in this paper.
